# Supplementary material for: Objective sleep quality in diverse older adults: a cross-sectional study on the importance of race and ethnicity and sex
Source: BMC Med. 2025 Jul 1;23:391. doi: 10.1186/s12916-025-04204-w (PMC12219036; doi:10.1186/s12916-025-04204-w)
Supplement: Supplementary file 1 — Additional file 1. Additional Methods. Covariates description. Table S1. Multivariable-adjusted means of objective continuous sleep measures by race and ethnicity. Table S2. Multivariable-adjusted associations between objective sleep measures and sociodemographic groups with further adjustment for household income. Table S3. Multivariable-adjusted means of objective continuous sleep measures by race and ethnicity with further adjustment for household income. Table S4. Multivariable-adjusted associations between objective sleep measures and sociodemographic groups with further adjustment for moderate to severe sleep apnea. Table S5. Multivariable-adjusted means of objective continuous sleep measures by race and ethnicity with further adjustment for moderate to severe sleep apnea. Table S6. Multivariable-adjusted means of objective continuous sleep measures by sex and age groups. Table S7. Multivariable-adjusted means of objective continuous sleep measures by sex and age groups with further adjustment for household income. Table S8. Multivariable-adjusted means of objective continuous sleep measures by sex and age groups with further adjustment for moderate to severe sleep apnea. Table S9. Objective categorical sleep measures by race and ethnicity and sex. Table S10. Description of covariates by race and ethnicity and sex. Table S11. Description of covariates by sex and age groups. Figure S1. Stepwise approach to identify most important contributors in the association between sleep duration and age groups. Figure S2. Stepwise approach to identify most important contributors in the association between sleep efficiency and age groups. Figure S3. Stepwise approach to identify most important contributors in the association between wake after sleep onset and age groups. Figure S4. Stepwise approach to identify most important contributors in the association between sleep fragmentation index and age groups. [file 12916_2025_4204_MOESM1_ESM.docx]

**ADDITIONAL FILE 1**

**Objective Sleep Quality in Diverse Older Adults: a Cross-sectional Study on the Importance of Race and Ethnicity and Sex**

Clémence Cavaillès, PhD, Katie L. Stone, PhD, Yue Leng, PhD, Carrie Peltz, PhD, Kristine Yaffe, MD

Additional file 1: Additional Methods

***Covariates***

Sociodemographic and health characteristics were self-reported through clinical interviews, and included age, sex, race and ethnicity, years of education, retirement status, household income, current smoking status, and medical history of hypertension, diabetes, stroke, heart attack, and depression. Body mass index (BMI) was calculated as the ratio of weight to height (kg/m^2^). Physical activity was measured using the Rapid Assessment of Physical Activity (RAPA) questionnaire and analyzed either as a continuous variable or dichotomized, with participants classified as physically active if their RAPA score was ≥6 [28]. Cognitive diagnoses were determined using an algorithmic decision tree and subsequently confirmed through a consensus review as normal control, mild cognitive impairment (MCI), and dementia [23]. Sleep medication use was assessed through a self-reported item from the Pittsburgh Sleep Quality Index: “During the past month, how often have you taken medicine (prescribed or “over the counter”) to help you sleep?” [29]. Responses were dichotomized into two groups: those who used sleep medications (less than once a week; once or twice a week; and three or more times a week) and those who did not (not during the past month). In a subsample (n=784), participants used the WatchPAT 200 (Itamar Medical Ltd., Caesarea, Israel) during one night for a home sleep apnea test. The respiratory event index (REI) was used to evaluate the presence of moderate to severe sleep apnea (REI ≥15).

Additional file 1: Table S1. Multivariable-adjusted means of objective continuous sleep measures by race and ethnicity (n=824).

|  | Black  (n=190) | MA  (n=282) | NHW  (n=366) |  |  |
| --- | --- | --- | --- | --- | --- |
|  | Adjusted means (95%CI) | Adjusted means (95%CI) | Adjusted means (95%CI) | *P* value | Direction |
| **Sleep duration**, hour | 7.0 (6.7,7.3) | 7.0 (6.7,7.4) | 7.3 (7.0,7.6) | 0.003* | NHW> B,MA |
| **Sleep efficiency**, % ^a^ | 86.0 (84.5,87.4) | 87.0 (85.5,88.5) | 88.8 (87.4,90.1) | <.0001* | NHW> B,MA |
| **WASO**, min ^b^ | 58.2 (50.5,67.0) | 53.6 (46.2,62.1) | 45.5 (39.5,52.5) | <.0001* | NHW< B,MA |
| **SFI**, % | 34.5 (31.8,37.2) | 29.2 (26.5,32.0) | 27.7 (25.0,30.3) | <.0001* | B> MA,NHW |

Abbreviations: CI, confidence interval; MA, Mexican American; NHW, Non-Hispanic White; SFI, sleep fragmentation index; WASO, wake after sleep onset

Models adjusted for race and ethnicity, sex, age, education, retirement status, cognitive impairment, body mass index, physical activity, smoking, sleep medications, and history of hypertension, diabetes, stroke, heart attack, and depression.

^a^ cube transformation; ^b^ log transformation; * results are significant after false discovery rate correction

Additional file 1: Table S2. Multivariable-adjusted associations between objective sleep measures and sociodemographic groups with further adjustment for household income (n=787).

|  | Black *versus* NHW | MA *versus* NHW |  |  | Women *versus*  Men |  |  | ≥65 years *versus*  <65 years |  |
| --- | --- | --- | --- | --- | --- | --- | --- | --- | --- |
|  | OR (95%CI) | OR (95%CI) | *P* value |  | OR (95%CI) | *P* value |  | OR (95%CI) | *P* value |
| **Sleep duration**, hour |  |  | 0.03* |  |  | 0.003* |  |  | 0.90 |
| <6 | 2.64 (1.38,5.04) | 1.56 (0.77,3.18) |  |  | 0.45 (0.27,0.73) |  |  | 1.14 (0.63,2.06) |  |
| [6-8] | 1 | 1 |  |  | 1 |  |  | 1 |  |
| >8 | 0.85 (0.51,1.44) | 0.79 (0.46,1.35) |  |  | 1.11 (0.74,1.68) |  |  | 1.06 (0.67,1.67) |  |
| **Sleep efficiency**, % |  |  | <.001* |  |  | 0.18 |  |  | 0.51 |
| ≥85 | 1 | 1 |  |  | 1 |  |  | 1 |  |
| <85 | 2.55 (1.56,4.16) | 1.84 (1.08,3.14) |  |  | 0.77 (0.52,1.13) |  |  | 0.86 (0.55,1.35) |  |
| **WASO**, min |  |  | 0.002* |  |  | 0.53 |  |  | 0.34 |
| <41.7 | 1 | 1 |  |  | 1 |  |  | 1 |  |
| [41.7-63.5] | 1.85 (1.10,3.10) | 1.48 (0.88,2.50) |  |  | 1.25 (0.84,1.86) |  |  | 1.02 (0.62,1.56) |  |
| >63.5 | 2.92 (1.72,4.96) | 1.72 (0.99,2.99) |  |  | 1.12 (0.74,1.70) |  |  | 1.01 (0.45,1.18) |  |
| **SFI**, % |  |  | <.0001* |  |  | 0.008* |  |  | 0.83 |
| <22.1 | 1 | 1 |  |  | 1 |  |  | 1 |  |
| [22.1-29.9] | 2.73 (1.57,4.76) | 1.10 (0.66,1.84) |  |  | 0.90 (0.60,1.35) |  |  | 0.93 (0.59,1.46) |  |
| >29.9 | 5.37 (3.08,9.36) | 1.37 (0.80,2.36) |  |  | 0.55 (0.36,0.83) |  |  | 1.07 (0.66,1.73) |  |

Abbreviations: CI, confidence interval; MA, Mexican American; NHW, Non-Hispanic White; SFI, sleep fragmentation index; WASO, wake after sleep onset

Models adjusted for race and ethnicity, sex, age, education, retirement status, cognitive impairment, body mass index, physical activity, smoking, sleep medications, and history of hypertension, diabetes, stroke, heart attack, depression, and household income.

* results are significant after false discovery rate correction

Additional file 1: Table S3. Multivariable-adjusted means of objective continuous sleep measures by race and ethnicity with further adjustment for household income (n=787).

|  | Black  (n=190) | MA  (n=282) | NHW  (n=366) |  |  |
| --- | --- | --- | --- | --- | --- |
|  | Adjusted means (95%CI) | Adjusted means (95%CI) | Adjusted means (95%CI) | *P* value | Direction |
| **Sleep duration**, hour | 6.9 (6.6,7.3) | 7.1 (6.7,7.4) | 7.3 (7.0,7.6) | 0.008* | NHW>MA>B |
| **Sleep efficiency**, % ^a^ | 86.4 (84.9,87.8) | 87.4 (85.8,88.8) | 88.9 (87.6,90.3) | <.0001* | NHW>MA,B |
| **WASO**, min ^b^ | 55.8 (48.3,64.5) | 51.7 (44.4,60.3) | 44.4 (38.4,51.3) | <.0001* | NHW< MA,B |
| **SFI**, % | 34.0 (31.2,36.7) | 28.7 (25.8,31.6) | 27.3 (24.6,30.0) | <.0001* | B> NHW,MA |

Abbreviations: CI, confidence interval; MA, Mexican American; NHW, Non-Hispanic White; SFI, sleep fragmentation index; WASO, wake after sleep onset

Models adjusted for race and ethnicity, sex, age, education, retirement status, cognitive impairment, body mass index, physical activity, smoking, sleep medications, and history of hypertension, diabetes, stroke, heart attack, depression, and household income.

^a^ cube transformation; ^b^ log transformation; * results are significant after false discovery rate correction

Additional file 1: Table S4. Multivariable-adjusted associations between objective sleep measures and sociodemographic groups with further adjustment for moderate to severe sleep apnea (n=784).

|  | Black *versus* NHW | MA *versus* NHW |  |  | Women *versus*  Men |  |  | ≥65 years *versus*  <65 years |  |
| --- | --- | --- | --- | --- | --- | --- | --- | --- | --- |
|  | OR (95%CI) | OR (95%CI) | *P* value |  | OR (95%CI) | *P* value |  | OR (95%CI) | *P* value |
| **Sleep duration**, hour |  |  | 0.04* |  |  | 0.002* |  |  | 0.97 |
| <6 | 2.31 (1.19,4.48) | 1.41 (0.69,2.89) |  |  | 0.46 (0.28,0.77) |  |  | 0.97 (0.52,1.80) |  |
| [6-8] | 1 | 1 |  |  | 1 |  |  | 1 |  |
| >8 | 0.76 (0.45,1.28) | 0.67 (0.40,1.14) |  |  | 1.30 (0.86,1.95) |  |  | 1.05 (0.66,1.67) |  |
| **Sleep efficiency**, % |  |  | 0.005* |  |  | 0.05 |  |  | 0.28 |
| ≥85 | 1 | 1 |  |  | 1 |  |  | 1 |  |
| <85 | 2.28 (1.38,3.76) | 1.68 (0.99,2.85) |  |  | 0.68 (0.46,1.00) |  |  | 0.78 (0.49,1.23) |  |
| **WASO**, min |  |  | 0.004* |  |  | 0.31 |  |  | 0.22 |
| <41.7 | 1 | 1 |  |  | 1 |  |  | 1 |  |
| [41.7-63.5] | 1.96 (1.17,3.30) | 1.66 (1.00,2.77) |  |  | 1.35 (0.91,2.00) |  |  | 0.94 (0.60,1.50) |  |
| >63.5 | 2.67 (1.56,4.58) | 1.78 (1.04,3.05) |  |  | 1.11 (0.74,1.67) |  |  | 0.68 (0.42,1.10) |  |
| **SFI**, % |  |  | <.0001* |  |  | 0.003* |  |  | 0.91 |
| <22.1 | 1 | 1 |  |  | 1 |  |  | 1 |  |
| [22.1-29.9] | 2.56 (1.46,4.50) | 0.90 (0.55,1.50) |  |  | 0.94 (0.63,1.41) |  |  | 1.10 (0.70,1.75) |  |
| >29.9 | 4.77 (2.71,8.39) | 1.31 (0.77,2.22) |  |  | 0.53 (0.35,0.79) |  |  | 1.05 (0.65,1.71) |  |

Abbreviations: CI, confidence interval; MA, Mexican American; NHW, Non-Hispanic White; SFI, sleep fragmentation index; WASO, wake after sleep onset

Models adjusted for race and ethnicity, sex, age, education, retirement status, cognitive impairment, body mass index, physical activity, smoking, sleep medications, and history of hypertension, diabetes, stroke, heart attack, depression, and moderate to severe sleep apnea.

* results are significant after false discovery rate correction

Additional file 1: Table S5. Multivariable-adjusted means of objective continuous sleep measures by race and ethnicity with further adjustment for moderate to severe sleep apnea (n=784).

|  | Black  (n=190) | MA  (n=282) | NHW  (n=366) |  |  |
| --- | --- | --- | --- | --- | --- |
|  | Adjusted means (95%CI) | Adjusted means (95%CI) | Adjusted means (95%CI) | *P* value | Direction |
| **Sleep duration**, hour | 6.9 (6.6,7.2) | 6.9 (6.6,7.3) | 7.2 (6.9,7.5) | 0.006* | NHW> MA,B |
| **Sleep efficiency**, % ^a^ | 86.1 (84.6,87.6) | 86.9 (85.4,88.4) | 88.6 (87.2,90.0) | <.0001* | NHW> MA,B |
| **WASO**, min ^b^ | 57.3 (49.4,66.4) | 53.4 (45.9,62.1) | 45.8 (39.6,53.1) | <.0001* | NHW< MA,B |
| **SFI**, % | 34.7 (31.9,37.4) | 29.6 (26.8,32.5) | 28.3 (25.5,31.0) | <.0001* | B> NHW,MA |

Abbreviations: CI, confidence interval; MA, Mexican American; NHW, Non-Hispanic White; SFI, sleep fragmentation index; WASO, wake after sleep onset

Models adjusted for race and ethnicity, sex, age, education, retirement status, cognitive impairment, body mass index, physical activity, smoking, sleep medications, and history of hypertension, diabetes, stroke, heart attack, depression, and moderate to severe sleep apnea.

^a^ cube transformation; ^b^ log transformation; * results are significant after false discovery rate correction

Additional file 1: Table S6. Multivariable-adjusted means of objective continuous sleep measures by sex and age groups (n=824).

|  | Men  (n=290) | Women  (n=534) |  |  | <65 years  (n=340) | ≥65 years  (n=484) |  |
| --- | --- | --- | --- | --- | --- | --- | --- |
|  | Adjusted means (95% CI) | Adjusted means (95% CI) | *P* value |  | Adjusted means (95% CI) | Adjusted means (95% CI) | *P* value |
| **Sleep duration**, hour | 7.0 (6.7,7.2) | 7.3 (7.0,7.6) | 0.0002* |  | 7.0 (6.8,7.3) | 7.2 (6.9,7.5) | 0.22 |
| **Sleep efficiency**, % ^a^ | 86.9 (85.5,88.2) | 87.7 (86.3,89.0) | 0.03* |  | 87.0 (85.6,88.3) | 87.6 (86.2,88.9) | 0.18 |
| **WASO**, min ^b^ | 52.2 (45.5,59.9) | 52.1 (45.5,59.7) | 0.98 |  | 53.8 (46.9,61.8) | 50.6 (44.0,58.1) | 0.18 |
| **SFI**, % | 32.2 (29.6,34.8) | 28.7 (26.2,31.3) | <.0001* |  | 30.5 (27.9,33.1) | 30.5 (27.8,33.1) | 0.99 |

Abbreviations: CI, confidence interval; SFI, sleep fragmentation index; WASO, wake after sleep onset

Models adjusted for race and ethnicity, sex, age, education, retirement status, cognitive impairment, body mass index, physical activity, smoking, sleep medications, and history of hypertension, diabetes, stroke, heart attack, and depression.

^a^ cube transformation; ^b^ log transformation; * results are significant after false discovery rate correction

Additional file 1: Table S7. Multivariable-adjusted means of objective continuous sleep measures by sex and age groups with further adjustment for household income (n=787).

|  | Men  (n=290) | Women  (n=534) |  |  | <65 years  (n=340) | ≥65 years  (n=484) |  |
| --- | --- | --- | --- | --- | --- | --- | --- |
|  | Adjusted means (95% CI) | Adjusted means (95% CI) | *P* value |  | Adjusted means (95% CI) | Adjusted means (95% CI) | *P* value |
| **Sleep duration**, hour | 7.0 (6.7,7.3) | 7.2 (6.9,7.5) | 0.002* |  | 7.0 (6.7,7.4) | 7.1 (6.8,7.4) | 0.37 |
| **Sleep efficiency**, % ^a^ | 87.2 (85.8,88.6) | 87.9 (86.5,89.3) | 0.07 |  | 87.3 (85.8,88.6) | 87.9 (86.5,89.2) | 0.16 |
| **WASO**, min ^b^ | 50.4 (43.8,57.9) | 50.5 (43.8,58.1) | 0.97 |  | 52.2 (45.4,60.1) | 48.7 (42.2,56.1) | 0.13 |
| **SFI**, % | 31.7 (29.1,34.3) | 28.2 (25.6,30.9) | <.0001* |  | 30.0 (27.3,32.6) | 30.0 (27.3,32.7) | 0.95 |

Abbreviations: CI, confidence interval; SFI, sleep fragmentation index; WASO, wake after sleep onset

Models adjusted for race and ethnicity, sex, age, education, retirement status, cognitive impairment, body mass index, physical activity, smoking, sleep medications, and history of hypertension, diabetes, stroke, heart attack, depression, and household income.

^a^ cube transformation; ^b^ log transformation; * results are significant after false discovery rate correction

Additional file 1: Table S8. Multivariable-adjusted means of objective continuous sleep measures by sex and age groups with further adjustment for moderate to severe sleep apnea (n=784).

|  | Men  (n=290) | Women  (n=534) |  |  | <65 years  (n=340) | ≥65 years  (n=484) |  |
| --- | --- | --- | --- | --- | --- | --- | --- |
|  | Adjusted means (95% CI) | Adjusted means (95% CI) | *P* value |  | Adjusted means (95% CI) | Adjusted means (95% CI) | *P* value |
| **Sleep duration**, hour | 6.9 (6.6,7.2) | 7.2 (6.9,7.5) | 0.0003* |  | 7.0 (6.7,7.2) | 7.1 (6.8,7.4) | 0.34 |
| **Sleep efficiency**, % ^a^ | 86.8 (85.4,88.2) | 87.6 (86.3,89.0) | 0.04 |  | 86.9 (85.4,88.2) | 87.6 (86.2,89.0) | 0.10 |
| **WASO**, min ^b^ | 51.9 (45.0,59.9) | 51.9 (45.2,59.8) | 0.99 |  | 54.0 (46.7,62.3) | 50.0 (43.3,57.7) | 0.10 |
| **SFI**, % | 32.6 (29.9,35.3) | 29.1 (26.5,31.7) | <.0001* |  | 30.9 (28.2,33.6) | 30.7 (28.0,33.4) | 0.84 |

Abbreviations: CI, confidence interval; SFI, sleep fragmentation index; WASO, wake after sleep onset

Models adjusted for race and ethnicity, sex, age, education, retirement status, cognitive impairment, body mass index, physical activity, smoking, sleep medications, and history of hypertension, diabetes, stroke, heart attack, depression, and moderate to severe sleep apnea.

^a^ cube transformation; ^b^ log transformation; * results are significant after false discovery rate correction

Additional file 1: Table S9. Objective categorical sleep measures by race and ethnicity and sex.

|  | Men  (n=295) | | | |  | Women  (n=543) | | | |
| --- | --- | --- | --- | --- | --- | --- | --- | --- | --- |
|  | Black  (n=59) | MA  (n=84) | NHW  (n=152) |  |  | Black  (n=131) | MA  (n=198) | NHW  (n=214) |  |
|  | No. (%) | No. (%) | No. (%) | *P* value^a^ |  | No. (%) | No. (%) | No. (%) | *P* value^a^ |
| **Sleep duration**, hour |  |  |  | 0.0008* |  |  |  |  | 0.002* |
| <6 | 18 (30.5) | 19 (22.6) | 14 (9.2) |  |  | 20 (15.3) | 23 (11.6) | 9 (4.2) |  |
| [6-8] | 31 (52.5) | 55 (65.5) | 101 (66.4) |  |  | 84 (64.1) | 131 (66.2) | 137 (64.0) |  |
| >8 | 10 (16.9) | 10 (11.9) | 37 (24.3) |  |  | 27 (20.6) | 44 (22.2) | 68 (31.8) |  |
| **Sleep efficiency**, % |  |  |  | <.0001* |  |  |  |  | <.0001* |
| ≥85 | 32 (54.2) | 52 (61.9) | 129 (84.9) |  |  | 92 (70.2) | 140 (70.7) | 189 (88.3) |  |
| <85 | 27 (45.8) | 32 (38.1) | 23 (15.1) |  |  | 39 (29.8) | 58 (29.3) | 25 (11.7) |  |
| **WASO**, min |  |  |  | <.0001* |  |  |  |  | <.0001* |
| <41.4 | 8 (13.6) | 25 (29.8) | 77 (50.7) |  |  | 33 (25.2) | 43 (21.7) | 93 (43.5) |  |
| [41.4-63.7] | 20 (33.9) | 26 (31.0) | 41 (27.0) |  |  | 44 (33.6) | 73 (36.9) | 76 (35.5) |  |
| >63.7 | 31 (52.5) | 33 (39.3) | 34 (22.4) |  |  | 54 (41.2) | 82 (41.4) | 45 (21.0) |  |
| **SFI**,% |  |  |  | <.0001* |  |  |  |  | <.0001* |
| <22.1 | 3 (5.1) | 24 (28.6) | 65 (42.8) |  |  | 25 (19.1) | 64 (32.3) | 99 (46.3) |  |
| [22.1-29.9] | 20 (33.9) | 18 (21.4) | 46 (30.3) |  |  | 42 (32.1) | 79 (39.9) | 73 (34.1) |  |
| >29.9 | 36 (61.0) | 42 (50.0) | 41 (27.0) |  |  | 64 (48.9) | 55 (27.8) | 42 (19.6) |  |

Abbreviations: MA, Mexican American; NHW, Non-Hispanic White; SFI, sleep fragmentation index; WASO, wake after sleep onset

^a^ Chi-square test was used
* results are significant after false discovery rate correction

Additional file 1: Table S10. Description of covariates by race and ethnicity and sex (n=838).

|  | Men  (n=295) | | | |  | Women  (n=543) | | | |
| --- | --- | --- | --- | --- | --- | --- | --- | --- | --- |
|  | Black  (n=59) | MA  (n=84) | NHW  (n=152) |  |  | Black  (n=131) | MA  (n=198) | NHW  (n=214) |  |
|  | No. (%) or mean (±SD) | No. (%) or mean (±SD) | No. (%) or mean (±SD) | *P* value^b^ |  | No. (%) or mean (±SD) | No. (%) or mean (±SD) | No. (%) or mean (±SD) | *P* value^b^ |
| Age, years | 63.1 (±6.4) | 65.9 (±8.0) | 71.9 (±8.0) | <0.0001 |  | 63.7 (±8.0) | 64.0 (±7.7) | 68.9 (±8.0) | <0.0001 |
| Age, *≥65 years* | 25 (42.4) | 42 (50.0) | 126 (82.9) | <0.0001 |  | 58 (44.3) | 94 (47.5) | 147 (68.7) | <0.0001 |
| Education, years | 14.1 (±2.4) | 10.8 (±4.4) | 16.4 (±2.7) | <0.0001 |  | 15.4 (±2.6) | 10.4 (±4.0) | 15.3 (±2.6) | <0.0001 |
| Retirement status | 33 (55.9) | 43 (51.2) | 114 (75.5) | 0.0003 |  | 67 (51.1) | 93 (47.2) | 157 (73.4) | <0.0001 |
| Income group |  |  |  | <0.0001 |  |  |  |  | <0.0001 |
| <25,000 | 20 (34.5) | 27 (34.6) | 13 (8.8) |  |  | 38 (29.9) | 90 (48.1) | 27 (13.3) |  |
| [25,000-50,000[ | 12 (20.7) | 23 (29.5) | 23 (15.6) |  |  | 25 (19.7) | 58 (31.0) | 52 (25.6) |  |
| [50,000-75,000[ | 7 (12.1) | 19 (24.4) | 29 (19.7) |  |  | 20 (15.7) | 17 (9.1) | 32 (15.8) |  |
| ≥75,000 | 19 (32.8) | 9 (11.5) | 82 (55.8) |  |  | 44 (34.6) | 22 (11.8) | 92 (45.3) |  |
| Cognitive impairment | 25 (42.4) | 31 (36.9) | 38 (25.0) | 0.03 |  | 34 (26.0) | 41 (20.7) | 19 (8.0) | <0.0001 |
| Smoke currently | 11 (18.6) | 8 (9.5) | 7 (4.6) | - |  | 9 (6.9) | 10 (5.1) | 5 (2.3) | - |
| RAPA score ^a^ | 4 (3-6) | 4 (3-6) | 4 (3-6) | 0.35 |  | 4 (3-6) | 3 (3-4) | 4 (3-6) | 0.0009 |
| BMI, kg/m^2^ ^a^ | 30.3 (26.5,36.2) | 29.6 (27.5,34.7) | 29.5 (25.6,32.9) | 0.22 |  | 32.5 (27.6,38.2) | 29.9 (26.7,35.3) | 28.8 (24.4,32.9) | <0.0001 |
| Sleep medications | 20 (33.9) | 27 (32.1) | 54 (35.5) | 0.87 |  | 47 (35.9) | 58 (29.3) | 101 (47.2) | 0.0008 |
| History of hypertension | 48 (81.4) | 58 (69.0) | 101 (66.4) | 0.10 |  | 101 (77.1) | 115 (58.1) | 114 (53.3) | <0.0001 |
| History of diabetes | 17 (28.8) | 30 (35.7) | 35 (23.0) | 0.11 |  | 40 (30.5) | 68 (34.3) | 20 (9.3) | <0.0001 |
| History of stroke | 1 (1.7) | 4 (4.8) | 8 (5.3) | - |  | 11 (8.4) | 3 (1.5) | 15 (7.0) | - |
| History of heart attack | 4 (6.8) | 5 (6.0) | 6 (4.0) | - |  | 6 (4.6) | 6 (3.0) | 6 (2.8) | - |
| History of depression | 14 (23.7) | 24 (28.6) | 37 (24.3) | 0.73 |  | 44 (33.6) | 84 (42.4) | 87 (40.7) | 0.25 |
| REI, *≥15* | 26 (52.0) | 44 (55.7) | 69 (48.3) | 0.56 |  | 64 (54.2) | 97 (51.3) | 99 (48.3) | 0.58 |

Abbreviations: BMI, body mass index; MA, Mexican American; NHW, Non-Hispanic White; RAPA, rapid assessment of physical activity; SD, standard deviation.

^a^ median (interquartile range) ^b^ Anova test was used for continuous variables with a normal distribution, Kruskal-Wallis test for continuous variables without a normal distribution and, Chi-square test for categorical variables

Additional file 1: Table S11. Description of covariates by sex and age groups.

|  | Men  (n=295) | Women  (n=543) |  |  | <65 years  (n=346) | ≥65 years  (n=492) |  |
| --- | --- | --- | --- | --- | --- | --- | --- |
|  | No. (%) or mean (±SD) | No. (%) or mean (±SD) | *P* value^b^ |  | No. (%) or mean (±SD) | No. (%) or mean (±SD) | *P* value^b^ |
| Race and Ethnicity |  |  | 0.003 |  |  |  | <.0001 |
| Black | 59 (20.0) | 131 (24.1) |  |  | 107 (30.9) | 83 (16.9) |  |
| MA | 84 (28.5) | 198 (36.5) |  |  | 146 (42.2) | 136 (27.6) |  |
| NHW | 152 (51.5) | 214 (39.4) |  |  | 93 (26.9) | 273 (55.5) |  |
| Sex, *women* | - | - | - |  | 244 (70.5) | 299 (60.8) | 0.004 |
| Age, years | 68.4 (±8.5) | 65.9 (±8.2) | <.0001 |  | 58.7 (±4.0) | 72.4 (±5.7) | <.0001 |
| Age, *≥65 years* | 193 (65.4) | 299 (55.1) | 0.004 |  | - | - | - |
| Education, years | 14.3 (±4.0) | 13.5 (±4.0) | 0.005 |  | 13.3 (±4.0) | 14.2 (±4.0) | 0.003 |
| Retirement status | 190 (64.6) | 317 (58.5) | 0.08 |  | 91 (26.4) | 416 (84.7) | <.0001 |
| Income groups |  |  | 0.001 |  |  |  | 0.57 |
| <25,000 | 60 (21.2) | 155 (30.0) |  |  | 92 (27.5) | 123 (26.4) |  |
| [25,000-50,000[ | 58 (20.5) | 135 (26.1) |  |  | 80 (24.0) | 113 (24.2) |  |
| [50,000-75,000[ | 55 (19.4) | 69 (13.3) |  |  | 45 (13.5) | 79 (17.0) |  |
| ≥75,000 | 110 (38.9) | 158 (30.6) |  |  | 117 (35.0) | 151 (32.4) |  |
| Cognitive impairment | 94 (31.9) | 94 (17.3) | <.0001 |  | 80 (23.1) | 108 (22.0) | 0.69 |
| Smoke currently | 26 (8.8) | 24 (4.4) | 0.01 |  | 27 (7.8) | 23 (4.7) | 0.06 |
| RAPA score ^a^ | 4 (3,6) | 4 (3,6) | 0.002 |  | 4 (3,6) | 4 (3,6) | 0.17 |
| BMI, kg/m^2^ ^a^ | 29.6 (26.3,33.5) | 30.1 (26.1,35.1) | 0.63 |  | 30.8 (26.7,36.6) | 29.3 (25.7,33.1) | <.0001 |
| Sleep medications | 101 (34.2) | 206 (37.9) | 0.29 |  | 130 (37.6) | 177 (36.0) | 0.64 |
| History of hypertension | 207 (70.2) | 330 (60.8) | 0.007 |  | 202 (58.4) | 335 (68.1) | 0.004 |
| History of diabetes | 82 (27.8) | 128 (23.6) | 0.18 |  | 89 (25.7) | 121 (24.6) | 0.71 |
| History of stroke | 13 (4.4) | 29 (5.3) | 0.55 |  | 13 (3.8) | 29 (5.9) | 0.16 |
| History of heart attack | 15 (5.1) | 18 (3.3) | 0.21 |  | 7 (2.0) | 26 (5.3) | 0.02 |
| History of depression | 75 (25.4) | 215 (39.6) | <.0001 |  | 131 (37.9) | 159 (32.3) | 0.10 |
| REI, *≥15* | 139 (51.1) | 260 (50.8) | 0.93 |  | 163 (50.0) | 236 (51.5) | 0.67 |

Abbreviations: BMI, body mass index; MA, Mexican American; NHW, Non-Hispanic White; RAPA, rapid assessment of physical activity; REI, respiratory event index; SD, standard deviation
^a^ median (interquartile range) ^b^ T-test was used for continuous variables with a normal distribution, Mann-Whitney U test for continuous variables without a normal distribution and, Chi-square test for categorical variables

Additional file 1: Figure S1. Stepwise approach to identify most important contributors in the association between sleep duration and age groups.


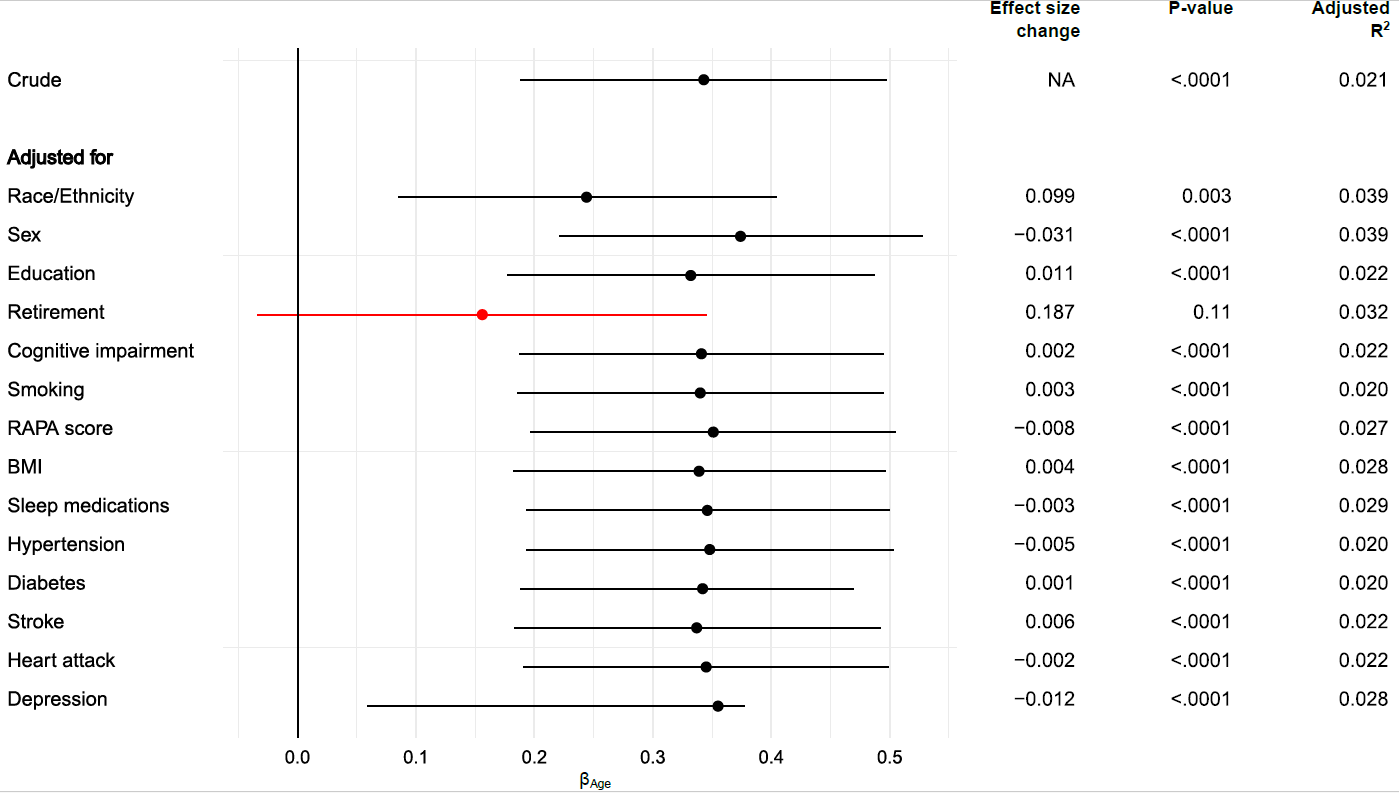


Abbreviations: BMI, body mass index; NA, not applicable; RAPA, rapid assessment of physical activity

Effect size change corresponds to the subtraction of the beta associated with the variable age in the crude model with the beta associated with age in each adjusted model.

Additional file 1: Figure S2. Stepwise approach to identify most important contributors in the association between sleep efficiency and age groups.


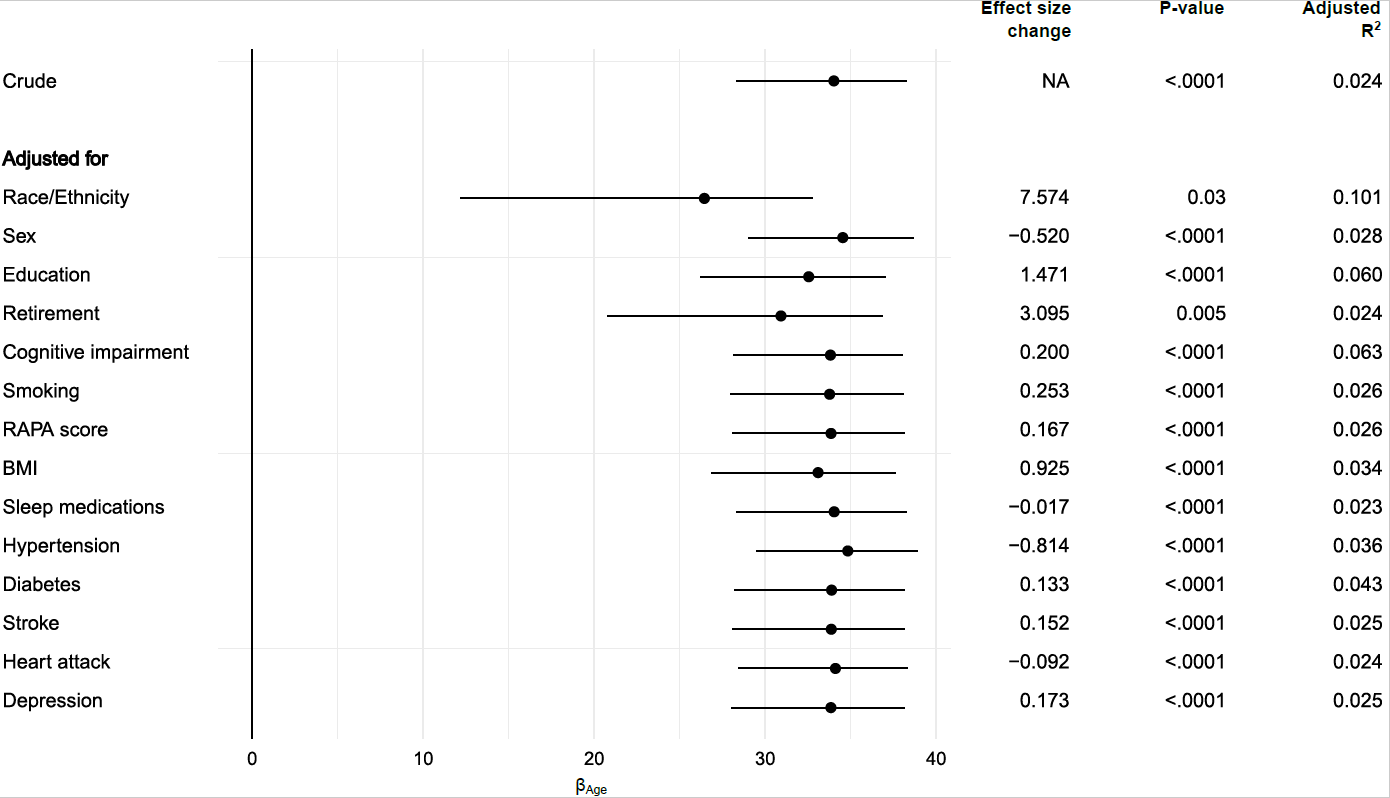


Abbreviations: BMI, body mass index; NA, not applicable; RAPA, rapid assessment of physical activity

Effect size change corresponds to the subtraction of the beta associated with the variable age in the crude model with the beta associated with age in each adjusted model.

Additional file 1: Figure S3. Stepwise approach to identify most important contributors in the association between wake after sleep onset and age groups.


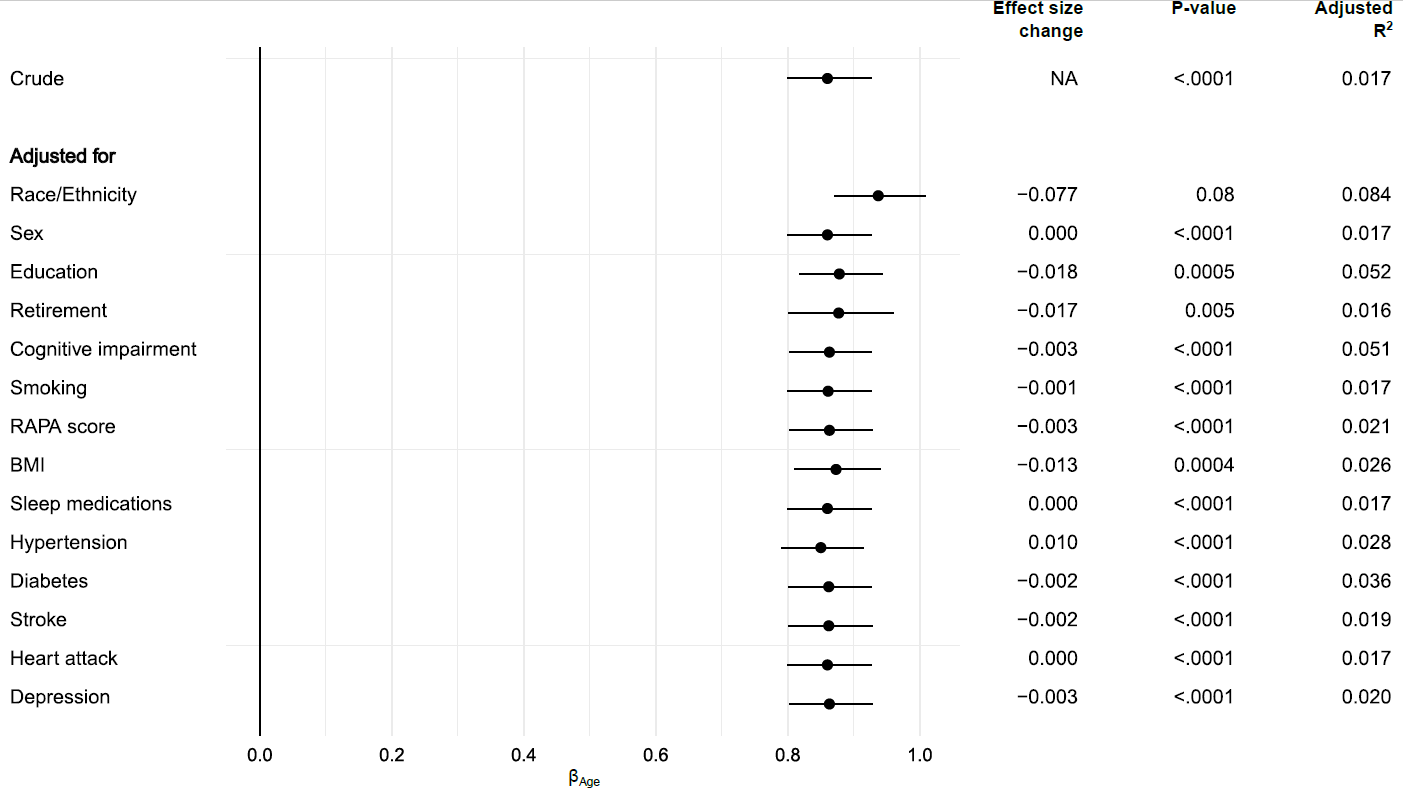


Abbreviations: BMI, body mass index; NA, not applicable; RAPA, rapid assessment of physical activity

Effect size change corresponds to the subtraction of the beta associated with the variable age in the crude model with the beta associated with age in each adjusted model.

Additional file 1: Figure S4. Stepwise approach to identify most important contributors in the association between sleep fragmentation index and age groups.


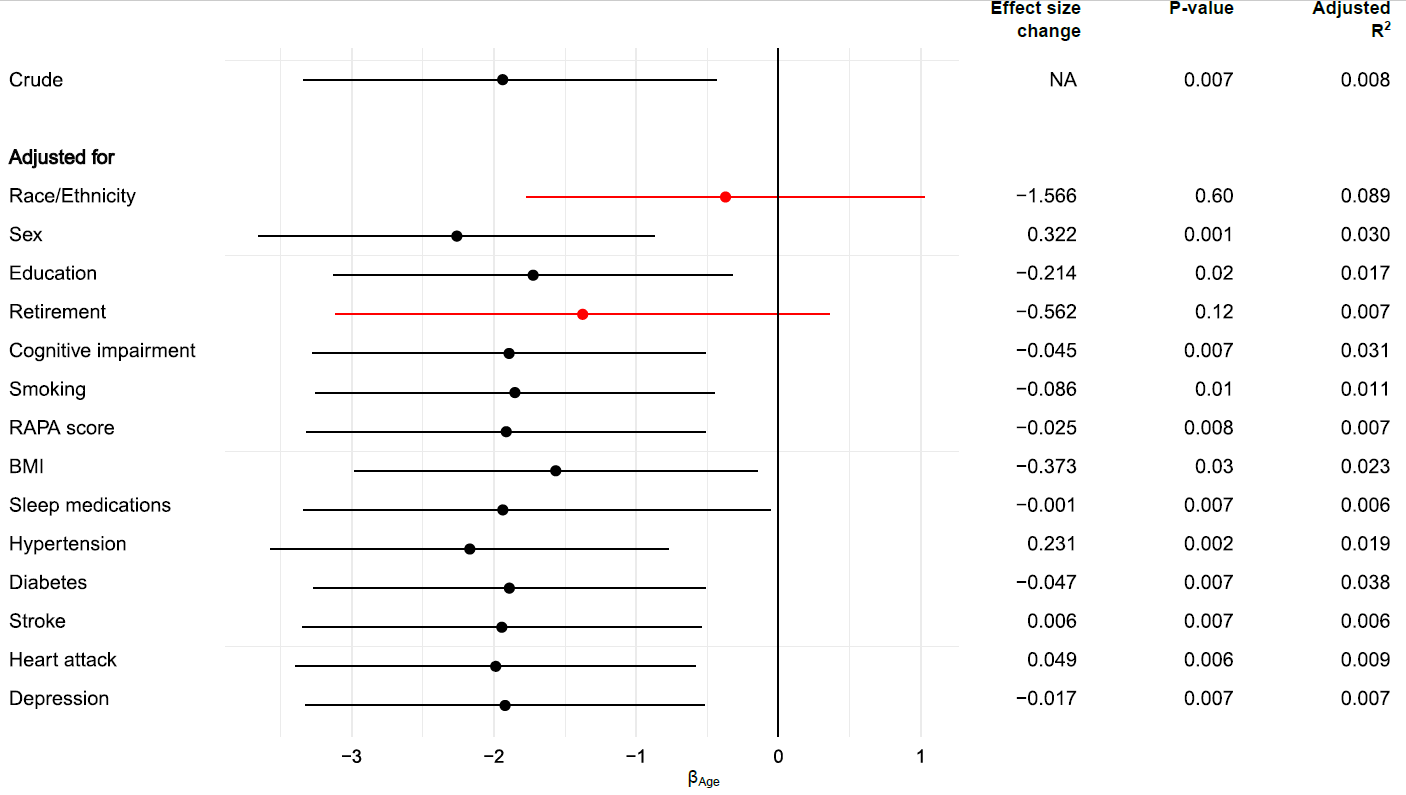


Abbreviations: BMI, body mass index; NA, not applicable; RAPA, rapid assessment of physical activity

Effect size change corresponds to the subtraction of the beta associated with the variable age in the crude model with the beta associated with age in each adjusted model.
